# Effect of shear bond strength in porcelain repair using five different surface treatments - An *in-vitro* study

**DOI:** 10.6026/973206300211973

**Published:** 2025-07-31

**Authors:** Aditya Vitthal Kulkarni, Mahesh Gandhewar, Nageshnath Baliram Waghmare, Ashish Diwakar Meshram, Mohit V Patil, Madhushree Patil

**Affiliations:** 1 Department of prosthodontics and crown and bridge, Government Dental College Jalgaon, Maharashtra, India; 2 Department of Prosthodontics and crown and bridge, ACPM Dental College Dhule, Thane, Maharashtra, India; 3 Dental Surgery, Government Medical College and Hospital Dharashiv, Maharashtra, India; 4 Department of Prosthodontics implantology crown and bridge, Shri Yashwantrao Chavan Memorial medical and Rural Development Foundation's Dental College and Hospital, Maharashtra, India

**Keywords:** Porcelain repair, shear bond strength, ceramic surface treatment, intraoral repair, composite resin bonding, Clearfil, Shofu, All Bond 2, hydrofluoric acid, diamond bur, sandblasting

## Abstract

The effect of surface treatments and bonding agents on the shear bond strength of composite resin to fractured metal-ceramic restorations is evaluated. Sixty Co-Cr discs were treated with diamond bur, sandblasting, hydrofluoric acid (HF), or their combinations and bonded using Clearfil, Shofu, or All Bond 2. Clearfil showed the highest bond strength, particularly with HF treatment. Shofu performed best with diamond + HF and All Bond 2 with sandblasting + HF. Thus, surface treatment and bonding agent selection significantly impact ceramic repair outcomes.

## Background:

Ceramics were introduced into dentistry as early as the 17th century, primarily for denture teeth. These materials are valued for their superior esthetics, chemical stability and mechanical properties such as compressive strength and abrasion resistance. However, they are also characterized by inherent limitations, including low tensile strength and high brittleness [1]. Despite continuous advancements in ceramic technologies and reinforcement strategies, ceramic veneer fractures remain a common clinical problem in metal-ceramic restorations. Studies report a 2-5% incidence of such fractures, typically at the metal-ceramic interface, making it the second most common cause for prosthesis replacement after secondary caries [2, 3]. Fractures in ceramic restorations, particularly in esthetically critical zones, impair both function and appearance. Complete replacement of prostheses is often expensive and time-consuming. Hence, intraoral repair using composite resins offers a minimally invasive and cost-effective alternative [4]. These repairs necessitate a strong and durable bond between the residual ceramic and the repair material to ensure long-term clinical success. Mechanical failures in metal-ceramic restorations may arise from trauma, improper occlusal design, Para functional habits, poor framework support, or mismatch in thermal expansion coefficients between the ceramic and metal components [5]. Common modes of ceramic failure include simple, mixed and complex fractures, with simple porcelain chipping being the most frequent [6]. The intraoral repair approach - typically involving surface conditioning followed by application of composite resins-can efficiently re-establish esthetics and functionality without removing the existing prosthesis [7]. Successful bonding of resin to ceramic surfaces involves both micromechanical interlocking and chemical adhesion. Surface treatments such as diamond bur abrasion, sandblasting with aluminum oxide particles and etching with hydrofluoric acid are employed to enhance surface roughness and promote micromechanical retention [8, 9]. Additionally, silane coupling agents and phosphate monomers such as 10-methacryloyloxydecyl dihydrogen phosphate (MDP) chemically bond to the ceramic matrix, improving adhesion between composite resins and silica-based ceramics [10]. Recent generations of porcelain repair systems have incorporated multipurpose bonding agents designed specifically for intraoral use. These systems integrate silane primers, adhesives and surface conditioners to simplify chairside procedures and improve bond durability under functional load [11]. Among commercially available products, Clearfil Porcelain Bond, Shofu Cera Resin Bond and All Bond 2 are commonly used for repairing fractured feldspathic ceramics, which are the standard materials for veneering metal substructures in prosthodontics [12]. Therefore, it is of interest to assess and compare the effectiveness of various surface treatments and bonding systems in enhancing the shear bond strength of composite resin to feldspathic porcelain, thereby optimizing intraoral repair outcomes in metal-ceramic restorations.

## Materials and Methods:

This *in-vitro* study aimed to assess the shear bond strength of ceramic repair systems following five different surface treatment protocols and using three commercially available bonding systems. A total of 60 samples were prepared for analysis.

## Sample fabrication:

Sixty disc-shaped porcelain specimens were fabricated using cobalt-chromium alloy as the metal framework. Each disc measured 1 cm in diameter and 0.5 mm in thickness. The porcelain was applied using a conventional layering technique and fired in a porcelain furnace. The surfaces of the specimens were smoothed using sandpaper.

## Grouping of samples:

The samples were randomly divided into five main groups (A-E) based on the type of surface treatment:

[1] Group A - Diamond bur abrasion

[2] Group B - Sandblasting with 100 µm aluminum oxide particles

[3] Group C - Etching with 5% hydrofluoric acid

[4] Group D - Combination of diamond bur + hydrofluoric acid

[5] Group E - Combination of sandblasting + hydrofluoric acid

Each of these five groups (n=12) was further subdivided into three subgroups (n=4) according to the bonding system used:

[1] Subgroup 1: Shofu Cera Resin Bond

[2] Subgroup 2: Clearfil Porcelain Bond

[3] Subgroup 3: All Bond 2 (Bisco)

## Surface treatment protocols:

[1] Group A: Abraded using a cone-shaped diamond bur (4138F) under air-rotor.

[2] Group B: Air abraded for 15 seconds using a sandblaster with 100 µm aluminum oxide.

[3] Group C: Etched with 5% hydrofluoric acid for 20 seconds.

[4] Group D: Treated with both diamond bur and 5% hydrofluoric acid etching.

[5] Group E: Treated with both sandblasting and 5% hydrofluoric acid etching.

## Bonding procedure:

Following surface treatment, a silane coupling agent was applied and allowed to dry. Each subgroup received one of the three bonding systems as per manufacturer instructions. Composite resin was incrementally applied and light-cured for a total of 200 seconds from five different angles.

## Thermocycling:

All samples were stored in distilled water for one week before thermocycling. A total of 500 thermocycles were performed using an automated thermocycler (Willytec, Germany), alternating between 5°C and 55°C to simulate oral conditions.

## Shear bond strength testing:

Samples were subjected to shear bond strength testing using a universal testing machine (Instron model 3345) with a crosshead speed of 0.5 cm/min and a 500 kg load cell. Load at failure was recorded and shear bond strength was calculated.

## Statistical analysis:

Data were analyzed using SPSS Version 25. Descriptive statistics included mean and standard deviation. Data normality was verified using the Shapiro - Wilk test. ANOVA was applied for intergroup and intragroup comparisons, followed by Tukey's post hoc test for pairwise significance. A p-value ≤ 0.05 was considered statistically significant.

## Results:

The present study aimed to evaluate and compare the shear bond strength of ceramic repair systems across five different surface treatments and three commercially available bonding agents. Sixty specimens were divided into 15 subgroups (n=4 each) based on the combination of surface treatment and bonding system. Table 1 (see PDF) summarizes the mean shear bond strength (in MPa) and standard deviation for each surface treatment applied in combination with the three bonding agents. Clearfil exhibited the highest bond strength following treatment with 5% hydrofluoric acid (20.82 ± 0.66 MPa), whereas All Bond 2 showed the lowest with sandblasting (10.69 ± 0.64 MPa).

Pairwise comparisons using Tukey's post hoc test showed that:

[1] For Clearfil, HF acid treatment led to significantly higher bond strengths compared to all other treatments (p < 0.001), with diamond bur and sandblasting groups showing the lowest.

[2] For Shofu, maximum bond strength was observed in the diamond bur + HF group (19.21 MPa), followed closely by the diamond-only group. Sandblasting alone resulted in significantly lower bond strength (12.62 MPa).

[3] For All Bond 2, the highest bond was seen in sandblasting + HF group (13.89 MPa), followed by the diamond + HF group (13.20 MPa), although overall values were significantly lower than the other two systems.

Significant differences were also observed across bonding agents within the same surface treatment:

[1] Under HF acid treatment, Clearfil showed superior bond strength (20.82 MPa), followed by Shofu and All Bond 2 (p < 0.001).

[2] With Diamond Bur + HF, Shofu recorded the highest bond strength (19.21 MPa), with significant differences from both Clearfil and All Bond 2 (p < 0.001).

[3] All Bond 2 consistently recorded the lowest bond strength across all surface treatments (Table 2, 3 - see PDF).

A two-way ANOVA revealed that both surface treatment (p < 0.001) and bonding agent (p < 0.001), as well as their interaction, had statistically significant effects on shear bond strength (p < 0.001).

## Discussion:

Metal-ceramic restorations have long been valued in restorative dentistry due to their high strength, esthetics and clinical longevity. Despite their success, veneering ceramic fractures remain a prevalent issue, often requiring repair or replacement. Full prosthesis replacement can be both financially and clinically demanding; hence, intraoral repair using composite-based systems presents a conservative and cost-effective solution, especially for minor fractures [1, 2]. The efficacy of ceramic repair systems is largely determined by the strength and durability of the bond between the fractured ceramic surface and the composite resin. Achieving such a bond requires both mechanical and chemical adhesion, often accomplished via surface pre-treatment [3]. Surface roughening-achieved through techniques such as diamond bur abrasion, sandblasting with aluminum oxide particles, or chemical etching using hydrofluoric acid-creates micromechanical interlocking sites that enhance resin infiltration and retention [4, 5]. In the present study, five surface treatment protocols were evaluated alongside three different commercially available bonding agents: Clearfil Porcelain Bond, Shofu Cera Resin Bond and All Bond 2. The results showed that the highest bond strength was achieved using Clearfil with hydrofluoric acid etching alone (20.82 MPa), while the combination of sandblasting and HF also performed well, indicating that acid etching plays a significant role in improving bonding efficacy (Table 1 - see PDF). These findings align with those of Estafan *et al.* who noted enhanced bond strength with combined mechanical and chemical conditioning of porcelain surfaces [6]. Shofu Cera Resin Bond performed best when used after a combination of diamond bur and HF etching, suggesting that this adhesive system benefits from deeper surface penetration facilitated by dual mechanical-chemical treatment [7]. All Bond 2, while yielding the lowest bond strength values among the three, still showed improved performance when paired with combined treatments rather than used alone. This supports the conclusions of Meshramkar *et al.* who emphasized that etching with 8% hydrofluoric acid + clearfil liner bond showed higher bond strength when compared to hydrofluoric acid alone [8]. Hydrofluoric acid etching consistently improved bond strength across all three bonding systems. This treatment increases surface porosity and creates a silica-rich layer, which reacts favorably with silane primers [9]. The use of a silane coupling agent enhances chemical bonding by forming siloxane bridges between the inorganic ceramic and the organic matrix of the resin [10]. Clearfil, which incorporates MDP monomers and silane into its formulation, likely outperformed others due to its optimized dual-bonding mechanism, consistent with findings by Bertolotti and colleagues [11]. Various studies have investigated the factors influencing the shear bond strength between restorative or orthodontic materials and porcelain or metal substrates. In general, porcelain surfaces have demonstrated superior bond strength compared to metal, particularly when metal surfaces are oxidized rather than machined, as oxidation enhances micromechanical retention and surface energy for bonding [12]. The use of silane coupling agents has been consistently shown to enhance adhesion, especially when applied to roughened porcelain surfaces, indicating the importance of surface pre-treatment in achieving durable bonds [13]. Among different primers and adhesion promoters, certain products such as Scotchprime Ceramic Primer have demonstrated notably higher bond strengths, highlighting the variability in performance among available bonding agents [14]. Furthermore, while the type of silane used can significantly affect bond strength, variations in hydrofluoric acid concentrations used for etching appear to have a lesser impact, suggesting that the chemical nature of the bonding agent plays a more critical role than the etching strength alone [15]. Despite promising findings, the current study's *in-vitro* nature poses limitations in mimicking true intraoral conditions. Parameters such as cyclic loading, salivary enzymes, pH variations and mechanical fatigue were not simulated beyond thermocycling. Further in-vivo studies are warranted to validate long-term clinical applicability of these systems.

## Conclusion:

The shear bond strength of ceramic repairs is significantly influenced by both the surface treatment method and the bonding system used. Among the bonding agents tested, Clearfil Porcelain Bond exhibited the highest bond strength, particularly when combined with hydrofluoric acid etching. The combination of mechanical (diamond bur or sandblasting) and chemical (HF) surface treatments consistently improved bond strength across all systems. Thus, selection of surface conditioning protocols and bonding agents can enhance the durability and success of intraoral ceramic repairs.
